# Enhancing the sensitivity of a micro-diaphragm resonating sensor by effectively positioning the mass on the membrane

**DOI:** 10.1038/srep17069

**Published:** 2015-11-23

**Authors:** Jinsik Kim, Hye Jin Kim, EunAe Cho, Hyun-Joon Shin, Jung Ho Park, Kyo Seon Hwang

**Affiliations:** 1 Center for BioMicrosystems, Korea Institute of Science and Technology, Seoul 136-791, Korea; 2 Department of Electrical Engineering, Korea University, Seoul 136-713, Korea; 3 Department of Materials Science and Engineering, Korea Advanced Institute of Science and Technology, Daejeon 305-701, Korea; 4 Center for Bionics, Korea Institute of Science and Technology, Seoul 136-791, Korea

## Abstract

The detection of biomarkers in the liquid phase using mechanical sensors is difficult because of noise caused by the liquid. To reduce and verify the side effects of liquid loading, we performed calculations and experiments to determine the shift in resonant frequency according to the loading conditions. A 2-μm-thick piezoelectric rectangular micro-diaphragm with a 500 × 500 μm membrane was used. These dimensions were determined such that there would be an analogous resonant frequency shift ratio in both (1, 1) and (2, 2) modes. By calculating and measuring the resonant frequency, we verified that the resonant frequency of the sensor would change only through contact with the liquid, even the resonant frequency change by only liquid much higher than the changes caused by the nanoparticles. The real signal constituted only 0.017% of the initial resonant frequency. To enhance the sensitivity by reducing the unexpected surface stress in the liquid, the liquid was dropped onto the surface of the micro-diaphragm. This resulted in an improvement of more than 10 times the sensitivity in both modes. In addition, by controlling the position in the micro-diaphragm resonating sensor, more sensitive positions with large displacements were determined according to each mode.

Piezoelectric-actuated resonating sensors are a promising and widely used type of sensors for detecting biomolecules or viscous media^1^,^2^,^3^,^4^,^5^,^6^. Each sensor has its own resonant frequency^7^. The detection of biomolecules or viscous media is based on the shift in resonant frequency that results from additional mass induced on the sensor, i.e., stress on the surface of the sensor; the resonant frequency by detection of biomolecules or viscous media is measured in liquid^8^,^9^. Therefore, it is necessary for the surface of the sensor to come into contact with the liquid for the sensor to detect biomolecules. This is challenging because any contact with a liquid media can significantly change the resonant frequency and reduce the quality of the measurement because of the damping effect of the liquid and the changes in the surface stress of the membrane, even without any reactions of the biomolecules^7^,^10^,^11^. The shift in resonant frequency owing to interaction with the biomolecules or viscous media is much lower than the noise level (shift by only liquid loading). Even if the loaded liquid dries without any reactions, the resonant frequency is shifted because of the contact with the liquid.

In our previous research, we examined sensing of the viscosity and density of blood by using a micro-diaphragm. Changes in the resonant frequency and quality factor resulted from loading the liquid onto the optimized loading side^12^. The study was, however, about the properties of the liquid and the highly optimistic case whereby the disadvantages of liquid detection were overcome by the ability to detect the properties of a liquid. In general, liquid-phase-based sensing has a lower sensitivity owing to the side effects of liquid loading. To reduce the hydroelastic influence and enhance the sensitivity of a micro-diaphragm, the addition of holes to the micro-diaphragm^13^,^14^ and the thickness mode of a micro-diaphragm^7^ have been considered through miniaturization. Efforts to reduce the surface stress have included using a nano-fluidic channel in a micro-cantilever^6^. Despite these versatile approaches in the use of various mechanical sensors, the contact of the sensors (especially the micro-diaphragm) with the liquid remains unchanged.

In this study, effective mass loading on the surface of a thin-film micro-diaphragm was introduced to enhance the sensitivity by reducing the unexpected surface stress from the liquid. A simple loading method can reduce the surface stress and enhance the sensitivity without additional fabrication steps for miniaturization. A 2-μm-thick piezoelectric rectangular micro-diaphragm resonating sensor, with a membrane measuring 500 × 500 μm was used. The dimensions were determined so as to have an analogous resonant frequency shift ratio in the (1, 1) and (2, 2) modes. The effect of water contact, which gives rise to noise, was also verified by calculating and measuring the resonant frequency in both cases of (1) liquid-loading only and (2) after drying. Positioning the liquid at a specific point on the membrane of the micro-diaphragm enhanced the sensitivity by a factor of ten, relative to the sensitivity when the entire surface was loaded. Notably, the more sensitive positions, for which the displacement was larger, were determined according to each mode.

## Results

**Figure 1:**
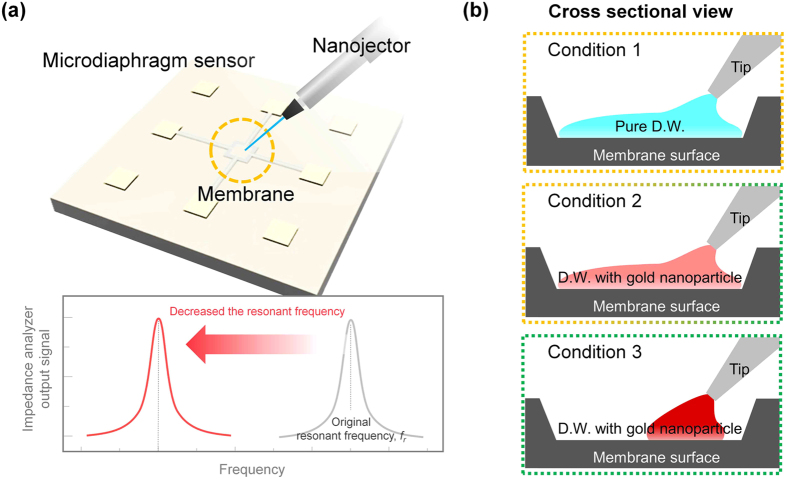
Schematic illustration of analysis of influence on and improvement of the sensitivity of a micro-diaphragm resonating sensor by effective positioning. (**a**) Scheme and basic principle of micro-diaphragm sensor. (**b**) Conditions under which hydroelastic influences are confirmed (Conditions 1 and 2) and for enhancing the sensitivity of the micro-diaphragm resonating sensor (Conditions 2 and 3).

Most biomolecules require a liquid phase environment, such as that provided by a buffer solution. Exposure of the surface of the sensor to a liquid is therefore essential for detecting biomolecules. The biomolecules in the liquid change the surface stress, causing a change in the resonant frequency of the micro-diaphragm sensor. The change in the resonant frequency is greater, however, because of the interaction with the liquid rather than the biomolecules on the surface. As shown in Fig. 1a, the side effects of loading the liquid media can also be observed with a micro-diaphragm resonating sensor. These effects are such that any real changes in the signal resulting from the interaction of biomolecules are indistinguishable from the overall changes. Figure 1 is a schematic illustration of the analysis of the influence and improvement of the sensitivity of a micro-diaphragm resonating sensor by effective positioning. The liquid is loaded under the surface of the micro-diaphragm by a nanojector tip (Fig. 1a) to reduce the influence of gravity. Three conditions are compared to verify the hydroelastic influence (Conditions 1 and 2 in Fig. 1b) and to enhance the sensitivity of the micro-diaphragm resonating sensor (Conditions 2 and 3 in Fig. 1b). As given by Condition 1 in Fig. 1b, pure deionized water (D. W.) was loaded to measure the influence of liquid loading. D. W. containing gold nanoparticles was also loaded to detect the shift in resonant frequency resulting from the interaction of the gold nanoparticles with the overall surface, as shown in Condition 2 in Fig. 1b. These gold nanoparticles are commonly used for a sandwich assay to enhance the mass effect in mechanical sensor applications^12^. Finally, D. W. with gold nanoparticles was loaded onto specific sites on the surface of the micro-diaphragm surface to verify the effect of the positioning, as shown in Condition 3 of Fig. 1b.

First, the diaphragm dimensions that were least affected by the loading of the liquid were calculated to effectively demonstrate the manner in which the sensitivity can be enhanced by positioning. The difficulty in isolating real changes by target materials from those caused by liquid loading was also verified by adopting a theoretical approach.

In general, changes in the resonant frequency occur as a result of immersion in a liquid or as a result of contact with a liquid. The ratio of the shift in resonant frequency to the initial resonant frequency (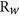) was calculated using equation (1) to verify the influence of water loading according to the dimensions of the diaphragm. Here, *f*_*i*_ and *f*_*w*_ are the natural resonant frequency of the micro-diaphragm without any loading and the shifted resonant frequency with loading on the surface. The value of *f*_*w*_ was calculated with added virtual mass incremental (AVMI) factors as shown in equation (2) for the (1, 1) mode and equation (3) for the (2, 2) mode^15^,^16^.


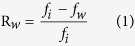



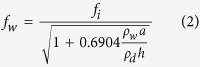



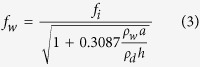


where*ρ*_*w*_, *ρ*_*d*_, *a*, and *h* are the densities of the water and the diaphragm layer, and the length and height of the diaphragm, respectively.

**Figure 2:**
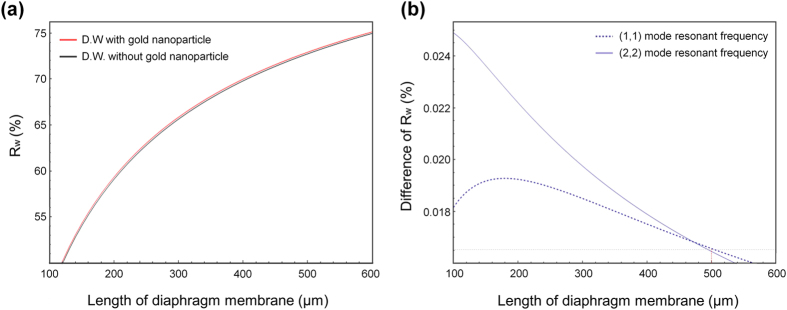
Calculated results for the ratio of the shift in resonant frequency. (**a**) Change in resonant frequency with length of diaphragm membrane under each condition, D. W. with and without gold nanoparticles. (**b**) Difference in resonant frequency according to dimensions of diaphragm membrane in both (1, 1) and (2, 2) modes.

The calculated values of R_w_ for the (1, 1) mode, according to the dimensions of the diaphragm, are shown in Fig. 2a. Greater changes in the resonant frequency occurred along the larger dimension of the diaphragm, when the liquid was loaded onto a micro-diaphragm having dimensions that could be fabricated, between 100 μm and 600 μm. The red and black solid lines shown in Fig. 2a show the values of R_w_ when D. W. alone and D. W. containing gold nanoparticles were loaded. The 10-nm diameter of the gold nanoparticles was adopted as a parameter for use in the following experiments. A significant change in the resonant frequency of over 60% was obtained with the water loading only case (black solid line). This water produced noise and interfered with the detection of the real changes caused by the gold nanoparticles. The narrow gap between the red and black lines—that is, the difference in R_w_—was the real change resulting from the gold nanoparticle loading. This clearly indicates the difficulty in detecting gold nanoparticles in the liquid phase, while the smaller dimensions of the micro-diaphragm have a smaller effect on the liquid loading. To optimize the dimensions of the diaphragm, the modal dependence of the sensitivity was also calculated. Figure 2b shows the differences in R_w_
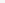
between the D. W. alone and the D. W. containing gold for the (1, 1) mode (solid line) and (2, 2) mode (dashed line). The difference in R_w_ represents the real change resulting from the gold nanoparticles in the liquid. As shown in Fig. 2b, larger changes occurred owing to the use of a micro-diaphragm with smaller dimensions, resulting in higher sensitivity. However, the differences in R_w_ between the modes ((1, 1) and (2, 2)) were quite different for smaller dimensions. The values are the same for long micro-diaphragms of about 500 μm, indicating that such a dimension could prevent modal dependence of the sensitivity. For this reason, a 500 × 500 μm micro-diaphragm was used for the ensuing experiments, as micro-diaphragms with smaller dimensions have a higher sensitivity. The difference in R_w_ of less than 0.017% was calculated such that the resonant frequency was shifted by several Hz in the presence of the gold nanoparticles. This also highlights the fact that the detection of gold nanoparticles in liquid is very difficult.

**Figure 3:**
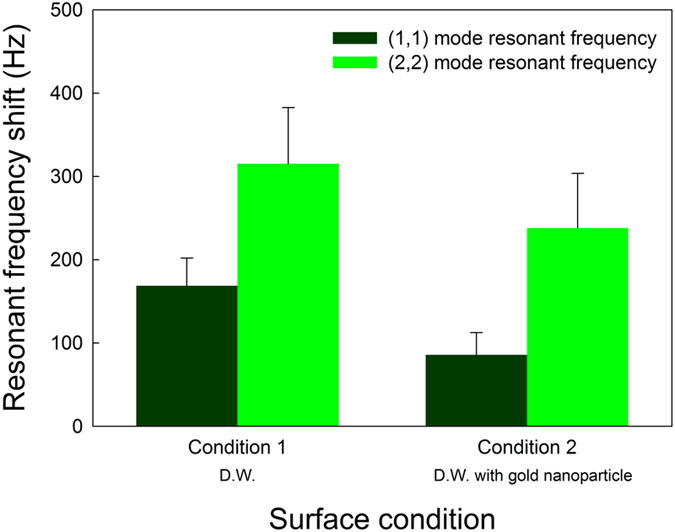
Change in resonant frequency according to loading of solution onto micro-diaphragm resonating sensor in both (1, 1) mode and (2, 2) mode.

The resonant frequency changes considerably as a result of contact with a liquid alone, without any reaction of the biomolecules. This change is greater than that of the real signal due to the change in the micro-diaphragm’s surface stress resulting from contact with the liquid. Therefore, the real signal is overwhelmed by the actual large signal. These effects are shown in Fig. 3. The D. W. without and with the gold nanoparticles were, respectively (Conditions 1 and 2 in Fig. 3) loaded onto the surface of the micro-diaphragm resonating sensor. The liquid was evaporated at room temperature overnight to enable the micro-diaphragm to become fully dry. The volume of the droplet placed on the surface was 1 μL and contained about 30 ng of gold nanoparticles. As mentioned earlier, the resonant frequency changed considerably when only D. W. was loaded (Condition 1) without any other mass and this significantly affected the resonant frequency. The values of the shift in resonant frequency for Condition 1 were 168.72 Hz and 315.31 Hz for the (1, 1) and (2, 2) modes, respectively. This result implies that contact with the liquid causes a noticeable change in the surface stress. The shift in resonant frequency for Condition 2, which was obtained with the D. W. with gold nanoparticles was 85.82 Hz and 238.12 Hz for both the (1, 1) and (2, 2) modes. The shift in resonant frequency (R_w_) with only the gold nanoparticles was 0.0366% and 0.01306% in both the (1, 1) and (2, 2) modes, as calculated using equation (2). The shift in resonant frequency was marginally smaller than the changes in Condition 1, despite the use of the D. W. containing the gold nanoparticles. In other words, the liquid loading was more influential than the mass of the gold nanoparticles. The slight difference in the resonant frequency between Conditions 1 and 2 was caused by the opposing effects of the surface stress and mass about the resonant frequency. In contrast to Condition 1, the resonant frequency changed as a result of loading with only D.W., with the resonant frequency being influenced by both the gold nanoparticles (mass) and the D. W. (surface stress) in Condition 2. These two factors simultaneously affected the resonant frequency and induced an opposing change in the resonant frequency. Therefore, the change in the resonant frequency was less than in Condition 1. More importantly, however, the real signal caused by mass loading was sufficiently minute that it did not induce a change in the resonant frequency. This debases the reliability and sensitivity of the micro-diaphragm. Consequently, the positioning method is suggested as a means of reducing the effects of the D. W. and enhancing the real signal.

The liquid could be locally located on the surface of the micro-diaphragm using a nanojector. This method is referred to as the “Positioning method” in this paper and can change the surface area affected by the stress (SAS) and mass (SAM). The real signal was enhanced by reducing the SAS and SAM.

**Figure 4:**
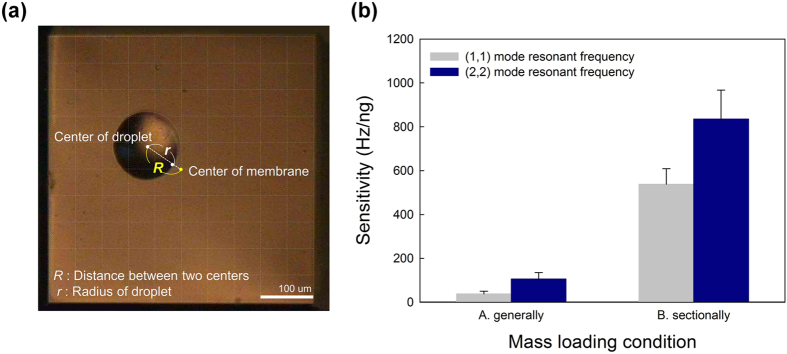
Shift in resonant frequency under each mass loading condition. (**a**) Microscope image of droplet on surface of the micro-diaphragm resonating sensor. (**b**) Change in resonant frequency according to mass loading condition.

Figure 4a shows a microscope image of the surface of the micro-diaphragm resonating sensor onto which D. W. with gold nanoparticles was dropped in a specific position. The position of the droplets is described as the distance (*R*) between the centre of the surface and the droplet, with the centre of the surface of the micro-diaphragm being set to 0 μm. The radius of the loaded droplet was *r* and the loaded mass of the gold colloid solution was calculated using *r* and the concentration of the solution. The loaded mass on the surface of the micro-diaphragm sensor could be controlled to adjust the mass between 0.28 ng (*r* is 25 μm) and 143.27 ng (*r* is 200 μm).

The resonant frequency was measured in two cases: in the first case, the D. W. with gold nanoparticles was spread over the entire surface of the micro-diaphragm sensor (“A. generally” in Fig. 4b) while in the second case the solution was dropped only onto part of the surface (“B. sectionally” in Fig. 4b). That is, the SAS and SAM in the latter case were smaller than in the former case.

The total loaded mass was 2.24 ng (*r* is 50 μm) in both cases. When the gold colloid solution was sectionally loaded onto the surface, the droplet was positioned in the centre of the micro-diaphragm sensor. Although the same mass was loaded onto the surface of the micro-diaphragm resonating sensor in both of these cases, the shift in resonant frequency and sensitivity proved to be extremely different. The sensitivity was defined as being the shift in resonant frequency per loaded mass. When the D. W. with the gold nanoparticles was loaded onto the entire surface, the sensitivity, as determined from the resonant frequency, changed by approximately 38.31 Hz/ng or 106.30 Hz/ng in both the (1, 1) mode and (2, 2) mode. Meanwhile, the sensitivity was about 262.01 Hz/ng or 277.22 Hz/ng when the gold colloidal solution was dropped onto the surface of the micro-diaphragm resonating sensor. This three- to seven-fold improvement in the sensitivity was attributed to the positioning method. The local loading of the gold solution onto the surface resulted in a decrease in the SAS and SAM. These decreases acted in opposition to the stress and mass. As a result, the decrease in SAS and SAM led to the mass being influential whereas the surface stress had no influence. As a result, the pointing method leads to a bigger change in the resonant frequency such that the performance of the micro-diaphragm resonating sensor can be improved. In addition, these focused effects occur differently according to the position of the droplets on the surface of the micro-diaphragm.

**Figure 5:**
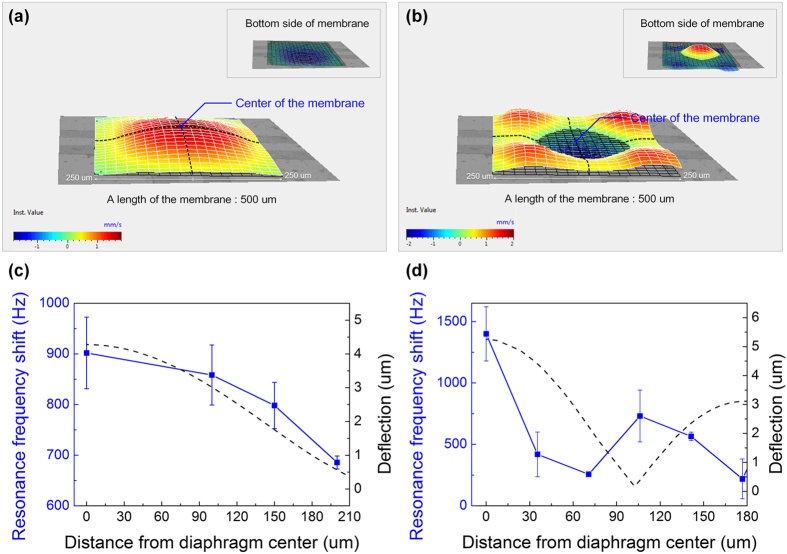
(**a,b**) Resonance of micro-diaphragm resonating sensor in both (1, 1) and (2, 2) modes, and (**c,d**) shift in resonant frequency of micro-diaphragm according to the distance from the centre of the micro-diaphragm sensor surface in both (1, 1) and (2, 2) modes.

To verify the effects of the position at which the gold colloid solution was loaded, and also confirm the most effective position, the sensitivity resulting from the position of the loaded droplet on the surface of the micro-diaphragm was also measured. Figure 5a,b show the resonating mode shape of the micro-diaphragm in both (1, 1) and (2, 2) modes. The insets show the resonating movement at the bottom of the micro-diaphragm resonating sensor. The micro-diaphragm resonating sensor can be displaced in one direction according to its position in the (1, 1) mode. Thus, the micro-diaphragm resonating sensor has one broad red colour region up to about 150 μm from the centre, while the colour changes beyond about 150 μm. This means that the surface exhibits the greatest deflection at the centre in the (1, 1) mode. In the (2, 2) mode, however, the micro-diaphragm resonating sensor is displaced in a different direction according to its position and has five small narrow red colour regions at centre and at each edge of the surface.

Figure 5c,d show the shift in resonant frequency of the micro-diaphragm according to the change in the distance between the droplet and the centre of the micro-diaphragm resonating sensor in both the (1, 1) mode and (2, 2) mode. The blue line represents the shift in resonant frequency owing to the droplet and the black dashed line represents the cross sectional mode shape (deflection) of the membrane according to the distance from the centre of micro-diaphragm resonating sensor. The radius and mass of the loaded droplet are approximately 50 μm and 2.24 ng, respectively. The shift in resonant frequency of the micro-diaphragm according to the droplet loading position exhibits a different tendency in each (1, 1) and (2, 2) mode and follows the mode shapes^17^,^18^,^19^. In the (1,1) mode, the resonant frequency of the micro-diaphragm resonating sensor changes the most at the centre, and the values reduce farther away from the centre of membrane. The resonant frequency of the micro-diaphragm changes by 901.92 Hz owing to the loaded droplet at the centre of the membrane; it changes by 685.64 Hz at 180 μm away from the centre. In addition, this change in frequency tendency perfectly matches with the deflection of the membrane, as shown in the Fig. 5c. The highest sensitivity of the micro-diaphragm resonating sensor is 402.65 Hz/ng, and it is obtained when a droplet is loaded onto the centre of the surface on the membrane.

The resonant frequency owing to the loaded droplet and its position change intricately in the (2, 2) mode because the resonating movement of the micro-diaphragm resonating sensor is more complex than that in the (1, 1) mode, as shown in Fig. 5d. In the (2, 2) mode, the resonant frequency has two peak points, in contrast to the (1, 1) mode. The resonant frequency owing to the droplet changed the most at the centre of the diaphragm membrane and decreased until about 70 μm away from the centre of membrane. However, the change in resonant frequency increased nearly 100 μm away from the diaphragm centre, after which it decreased again. These tendencies regarding the change in the resonant frequency is a result of the deflection of membrane according to the distance from the centre of micro-diaphragm resonating sensor. The change in resonant frequency and the sensitivity have maximum values of 1400.55 Hz and 625.25 Hz/ng, respectively. The change in resonant frequency and the sensitivity have values of 730.85 Hz and 326.27 Hz/ng at approximately 106 μm from the centre of micro-diaphragm resonating sensor.

These results demonstrate that focused stress causes a more significant change in the resonant frequency than normally induced stress over the entire surface of the micro-diaphragm sensor. Although the same mass is loaded onto the surface of the micro-diaphragm resonating sensor, the resonant frequency changes with the position at which the mass is loaded. Furthermore, the most effective position was confirmed according to the mode of the micro-diaphragm resonating sensor. Consequently, by adjusting the position at which the mass is loaded, we can improve the sensitivity of the micro-diaphragm resonating sensor.

## Discussion

In summary, the side effects of liquid loading were verified and enhancement of the sensitivity of mechanical micro-diaphragm sensors was demonstrated by local positioning to realize detection of biomarkers in the liquid phase. Calculations and experiments were employed to determine the changes due to the loading conditions with 2-μm-thick piezoelectric layer embedded micro-diaphragm with a 500 × 500 μm membrane. Our goal is to make local positioning the most effective means of detecting real biomaterials in the liquid phase without reducing the size of the sensors such as nano-scale reducing sensors, which are very costly and have a low fabrication yield. The improvement acquired in sensitivity was more than 10 times higher in both (1, 1) and (2, 2) modes. Furthermore, the most effective positions for both (1, 1) and (2, 2) modes were also determined. However, there are also bottlenecks limiting the further improvement of the analytical models of effective positioning, sensitivity and accuracy of the local positioning. An accurate analytic model is needed for accurate and effective positioning of mass loading^17^,^18^,^19^. A method of attaining smaller and more accurate drops than those that we demonstrated would result in a higher level of sensitivity and accuracy. Evaporation is also important. Natural evaporation occurred easily given the tiny volume of liquid used for real bio-application. Biomolecules that are highly susceptible to the condition of the liquid media, such as exosome and proteins, require that the conditions provided by the surrounding media be maintained.

With these further considerations of the effective positioning of mass loading, we believe that micro-diaphragm mechanical sensors will be adopted in different fields such as in the diagnosis of specific diseases, and the detection of hazardous liquids and gases.

## Methods

### Chemicals

A 10-nm gold colloid solution was purchased from Life Technologies^TM^. The standard concentration of the solution was 30 μg/mL.

### Fabrication of the micro-diaphragm resonating sensor

The micro-diaphragm resonating sensor was fabricated using a MEMS process. The micro-diaphragm resonating sensor consisted of SiO_2_/Pt/PZT/Pt/SiN_x_ layers with a PZT layer to enable self-actuation. The dimensions of the micro-diaphragm resonating sensor were 500 × 500 μm, and it was 3.45 μm thick. The resonant frequency in both the (1, 1) mode and (2, 2) mode was measured, in kHz.

### Micro-diaphragm resonating sensor experimental setup

To determine the positioning on the membrane of the micro-diaphragm resonating sensor, an impedance analyser (Agilent 4294A, Agilent Technologies) and a nanojector (Nanoject II^TM^, Drummond scientific company) were used. The microscope was focused on the surface of the micro-diaphragm resonating sensor and the nanojector dropped the liquid onto the surface under the command of a nanojector controller. The impedance analyser measured the resonant frequency in both (1, 1) and (2, 2) modes before and after complete loading of the droplet. By controlling the injection speed and time, the size of the droplet could be adjusted. All the experiments were progressed in 25 °C temperature and 30% humid laboratory.

### MSA-500 Micro System Analyzer

The resonance of the micro-diaphragm according to the mode was observed using an MSA-500 Micro System Analyzer (Polytec Inc.).

## Additional Information

**How to cite this article**: Kim, J. *et al.* Enhancing the sensitivity of a micro-diaphragm resonating sensor by effectively positioning the mass on the membrane. *Sci. Rep.* **5**, 17069; doi: 10.1038/srep17069 (2015).
